# Comparison of the methods for profiling *N*-glycans—hepatocellular carcinoma serum glycomics study†

**DOI:** 10.1039/c8ra02542h

**Published:** 2018-07-20

**Authors:** Ran Wang, Yufei Liu, Chang Wang, Henghui Li, Xin Liu, Liming Cheng, Yanhong Zhou

**Affiliations:** Britton Chance Center for Biomedical Photonics at Wuhan National Laboratory for Optoelectronics – Hubei Bioinformatics & Molecular Imaging Key Laboratory, Systems Biology Theme, Department of Biomedical Engineering, College of Life Science and Technology, Huazhong University of Science and Technology Wuhan 430074 China yhzhou@hust.edu.cn xliu@mail.hust.edu.cn; Department of Laboratory Medicine, Tongji Hospital Wuhan 430074 China chengliming2002@163.com

## Abstract

Monitoring serum glycomics is one of the most important emerging approaches for diagnosis of various cancers, and the majority of previous studies were based on MALDI-MS or HPLC analysis. Considering the difference of these analytical methods employed for serum glycomics, it is necessary to compare the effectiveness of different analytical methods for monitoring the aberrant changes in serum glycomics. In this study, a strategy based on machine learning was firstly applied for comparing the analysis results of MALDI-MS and HPLC on the same serum glycomics of hepatocellular carcinoma (HCC) samples. The capability of these two analytical methods for identifying HCC is demonstrated by the classification results obtained from MALDI-MS and HPLC data. In addition, by comparing glycomics which were significantly correlated with HCC based on MALDI-MS and HPLC, some *N*-glycans which may be the potential biomarkers for HCC were identified, validating the capability of these two analytical methods for the differentiated identification in the analysis of glycomics. Meanwhile, it is noteworthy that various physiological and environmental factors may cause the aberrant changes in glycosylation, and all these interference factors may be minimized by analyzing the same sample sets of HCC. Overall, these results showed that MALDI-MS and HPLC are complementary in qualitative and quantitative analysis of serum glycomics.

## Abstract 2

Monitoring serum glycomics is one of the most important emerging approaches for diagnosis of various cancers, and the majority of previous studies were based on MALDI-MS or HPLC analysis.

## Graphical abstract

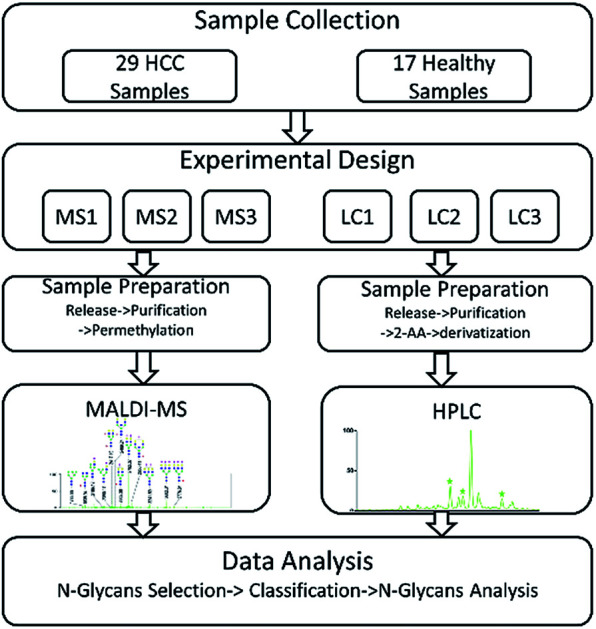

## 1. Introduction

Protein glycosylation is one of the most widespread post-translational modifications, playing crucial roles in many biological processes.^1–3^ Numerous cancer-related processes including oncogenic transformation,^3,4^ cancer progression,^5^ and antitumor immunity^6^ are associated with the aberrant glycosylation of proteins. Furthermore, various cancer markers are glycoproteins with alterations in serum glycomics.^7–9^ Due to the importance of glycosylation to biological processes, effective analytical methods for monitoring these aberrant changes in glycosylation are indeed required.

Various approaches for qualitative and quantitative analysis of subtle changes in glycomics mainly rely on several different analytical techniques, including high performance liquid chromatography (HPLC), capillary electrophoresis (CE), and mass spectrometry (MS) *etc.*^10^ Among those techniques, HPLC-fluorescent detection (HPLC-FLD) has become an effective means to analyze glycans, coupling with different fluorescent labelling reagents, such as 2-aminobenzoic acid (2-AA), 2-aminobenzamide (2-AB), and 2-amino pyridine (PA) *etc.*.^11^ Additionally, matrix-assisted laser desorption/ionization mass spectrometry (MALDI-MS) has also widely been applied to identify *N*-glycan biomarkers for cancer due to its low sample consumption, high throughput capacity and ease of operation.^12–14^

It is noteworthy that the *N*-glycomics of some cancers have been studied by MALDI-MS and HPLC, respectively.^1^ In these studies, both MALDI-MS and HPLC have identified several *N*-glycans as potential biomarkers for these cancers. Kyselova *et al.* reported that 8 *N*-glycans have been identified with highly accurate diagnostic potential in breast cancer by MALDI-MS,^15^ while in the study of Saldova *et al.*, another 4 different types of *N*-glycans have been identified as potential biomarkers for breast cancer by HPLC, and there was no overlap between these two studies in terms of the potential biomarkers.^16^ Additionally, Wu *et al.* have reported that 6 *N*-glycans were highly in correlation with lung cancer by MALDI-MS analysis,^17^ while in the study of Rudd *et al.*, 20 *N*-glycans have been identified as potential biomarkers for lung cancer by HPLC,^18^ and it should be noted that 3 common *N*-glycans were identified as significantly changed in lung cancer by both MALDI-MS and HPLC. However, those differences of *N*-glycans as potential biomarkers for these cancers by both two analytical methods hasn't been investigated in the same sample set. In addition, some common lifestyle parameters such as age, diet, smoking, body fat and plasma lipid status may cause the changes in glycosylation.^19–21^ And it also should be noted that some other factors including mutations of genes, different levels of cholesterol and insulin may also have effects on the aberrant glycosylation even for normal plasma glycomic profiles.^22^ Therefore, it is necessary to eliminate these interference factors between different sample sets which may lead to the variances in glycosylation, in prior to the appropriate evaluation of the identified *N*-glycans by these two analytical methods.

**Fig. 1:**
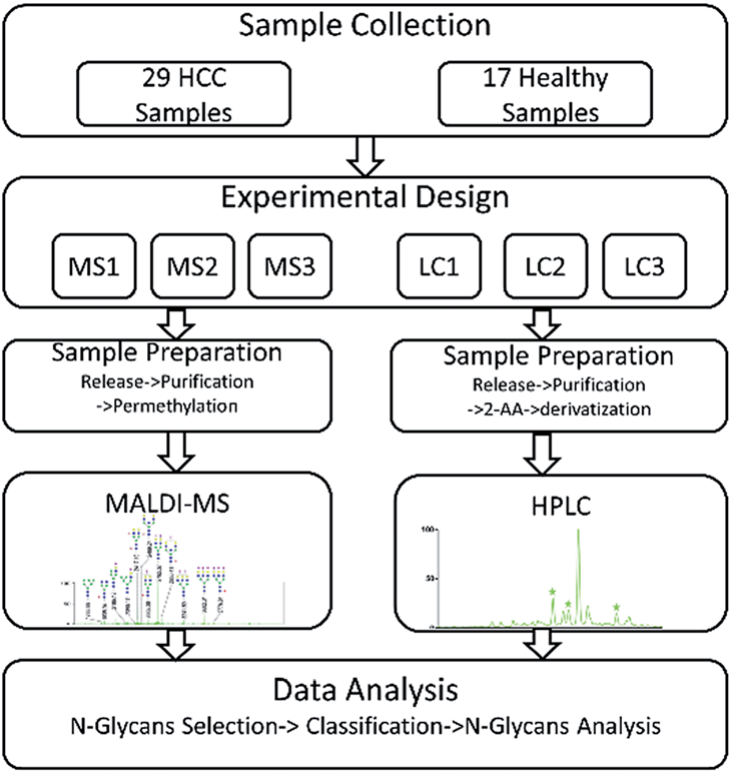
Workflow for comparison analysis of serum glycomics by MALDI-MS and HPLC in HCC.

In our study, HCC which is a common malignant disease with five-year relative survival rates less than 15%^10,11^ was selected to further investigate the internal factors. In order to eliminate the interference caused by different samples, two sets of same samples including HCC cases and healthy controls were derivatized and then analyzed by MALDI-MS and HPLC respectively. The workflow of the analytical process was shown in Fig. 1 and some *N*-glycans which were highly in correlation with HCC were identified. By statistical analysis, the difference in identification of glycoforms by MALDI-MS and HPLC were evaluated, further revealing the difference of these two analytical methods in biomarker discovery for HCC. Meanwhile, the relevance in biomarker discovery for HCC by MALDI-MS and HPLC was also explored, suggesting the complementary of these two analytical methods in qualitative and quantitative analysis of serum glycomics.

## 2. Materials and methods

### 2.1 Experimental section

#### 2.1.1 Reagent

Sodium hydroxide beads (20–40 mesh), porous-graphitized carbon (PGC), 2,5-dihydroxybenzoic acid (DHB) and 2-aminobenzoic acid (2-AA) were purchased from Sigma-Aldrich (MO, U.S.A.). *N*-glycosidase F (PNGase F) and endoglycosidase buffer pack (EBP) were purchased from New England Biolabs (MA, U.S.A.). Acetonitrile, (ACN) and methanol were from Merck KGaA (Darmstadt, Germany). Formic acid (FA) and pure water were obtained from Thermo Fisher Scientific (MA, U.S.A.). Trifluoroacetic acid (TFA), chloroform, sodium acetate and sodium chloride were from Sinopharm Chemical Reagent (Shanghai). Dimethyl sulfoxide (DMSO) was purchased from Aladdin Industrial Corporation (Shanghai). Iodomethane was obtained from Ai Keda Chemical Technology (Chengdu). Empty spin column and sample tube were purchased from Harvard Apparatus (U.S.A.). Human serum samples, including 29 HCC cases and 17 healthy controls, were donated by Tongji Hospital (Tongji Medical College, Huazhong University of Science and Technology). The study was carried out in accordance with the Helsinki declaration and informed consents were obtained from the participants in accordance with the study protocols approved by the Ethics Committee of Huazhong University of Science and Technology.

#### 2.1.2 Sample preparation

Serum sample of 10 μL was dissolved in 90 μL of solution containing 20 mM sodium phosphate (pH = 7.5), 0.13% dodecyl sulfate sodium and 10 mM dithiothreitol. The solution was incubated at 100 °C for 10 min prior to adding 12 μL of 10% octylphenoxypolyethoxyethanol (NP-40). The reaction mixture was then incubated with PNGase F at 37 °C for 18 h. After digestion, the sample was purified using PGC cartridge and dried in a vacuum concentrator (Eppendorf, Germany).

#### 2.1.3 Permethylation and purification of *N*-glycans

*N*-glycans released from serum samples were permethylated by a solid-phase protocol.^23^ Briefly, a spin column was packed by sodium hydroxide mesh beads which have been soaked with 200 μL DMSO, then centrifuged for 1 min at 1000 rpm to remove DMSO. The sample was dissolved in 35.9 μL of solution including 0.3 μL pure water, 30 μL DMSO and 5.6 μL iodomethane, and then applied to the prepared reaction spin column. After 25 min of incubation, extra 20 μL of iodomethane was added to the spin column. After another 15 min of incubation, the permethylated *N*-glycans were eluted using 200 μL of acetonitrile. Then the sample was dried in a vacuum concentrator.

Dried sample was dissolved in 800 μL of solution including 400 μL chloroform and 400 μL NaCl solution, then the extraction solution was mixed and incubated at room temperature for 20 min. Centrifuged the extraction solution for 1 min at 10 000 rpm and removed the supernatant. Extra 400 μL of pure water was added. After mixing for 1 min, remove the supernatant again and dried the sample in a vacuum concentrator.

#### 2.1.4 Fluorescence labeling and purification of *N*-glycans

The fluorescence labeling of *N*-glycans with 2-AA was conducted as following procedures: glycans were mixed with 20 μL reaction solution (48 mg mL^−1^ 2-AA in DMSO containing 30% acetic acid) and 20 μL reducing agent (1 M 2-picoline-borane in DMSO), then incubated at 65 °C for 3 h. It is noteworthy that the derivative reagent must be freshly prepared in labeling process.

The reaction mixture was purified by MCC cartridges as follows: MCC cartridges were equilibrated by 3.0 mL of 1-butanol/ethanol/H_2_O (4 : 1 : 1, v/v/v). After equilibrium, the derivatives were loaded on MCCs and washed with 3.0 mL of equilibrium solution. Finally, glycans were eluted by 1.0 mL of ethanol/H_2_O (1 : 1, v/v), and then dried by concentrator under vacuum.

#### 2.1.5 Analysis of permethylated *N*-glycans

The permethylated *N*-glycans were dissolved in 10 μL of 50% ACN. Then 0.5 μL of DHB and 0.5 μL of sample solution were mixed and spotted onto the stainless steel MALDI plate. The detection of the samples was accomplished by MALDI 4800 and the related system parameters were enumerated as follows: detection mode (reflector positive), laser intensity (5200 shots), detector voltage multiplier (0.86), and mass range (1300–5000 *m*/*z*). Meanwhile, all the *N*-glycan species observed by MALDI-MS were listed in Table S1 (ESI†), covering all the basic oligosaccharide types. The compositions of the *N*-glycans were abbreviated by [*a-b-c-d-e*] and the detailed information for nomenclature was shown in Scheme 1 (ESI†). The obtained MALDI-MS data were processed with Data Explorer 4.5. Smoothing step was performed with Gaussian smooth (filter width: 5 points). Data was further processed to generate .txt files listing *m*/*z* values and intensities (from ASCII Spectrum).

#### 2.1.6 Analysis of 2-AA-derivatized *N*-glycans by HPLC

The 2-AA-derivatized *N*-glycans were analyzed on a Shimadzu LC-20 AD separations module (Shimadzu, Milford, MA) equipped with a Shimadzu temperature control module and a Shimadzu RF-10A XL fluorescence detector. The separation of derivatized *N*-glycans was conducted on a TSK-Gel Amide-80 column (Tosoh, Bioscience Shanghai Co, LTD; 4.6 mm i.d., 250 mm) at 30 °C with a linear gradient consisted of 50 mM ammonium formate (pH 4.4) as solvent A and ACN as solvent B at a flow rate of 1.0 mL min^−1^. A 68 min run was used as keeping solvent B at 67.5 for 4 min and then the linear gradient of 67.5 to 53% solvent B over 59 min, followed by 1 min at 53 to 0% B and 3 min at 0% B, returning to 67.5% B over 1 min. The excitation/emission wavelengths of fluorometric detection were *λ*_ex_ = 360 nm and *λ*_em_ = 419 nm for 2-AA derivatives, respectively.

In order to confirm the chemical compositions of 2-AA derivatized *N*-glycans, the collections of each peak from HPLC were further analyzed by nanoLC-ESI-MS (AB SCIEX, USA) with C18 as solid phase (75 μm i.d. × 100 mm long, 5 μm; Proteomics Front, China). Solvent A was consisted of 5% ACN solution containing 0.1% FA (v/v), and solvent B was consisted of 95% ACN solution containing 0.1% FA (v/v). The injection volume for each collection was 2 μL. The solutes were eluted at a flow rate of 300 nL min^−1^ with gradient profile as follows: 95% to 95% A, 0 min; 95% to 90% A, 0 to 2 min; 90% to 70% A, 2 to 10 min; 70% to 40% A, 10 to 15 min; 40% to 5% A, 15 to 18 min; 5% to 5% A, 18 to 23 min; 5% to 95% A, 23 to 25 min; 95% to 95% A, 25 to 40 min. Data acquisition was conducted using an ion source gas of 3 PSI, a curtain gas of 35 PSI, an ion spray voltage of 2.3 kV, an interface heater temperature of 150 °C, and a collision energy of 10 eV for collision-induced dissociation (CID). MS was operated in the positive-ion mode with a mass range of 100–3000 *m*/*z*, and MS/MS was acquired in the information dependent acquisition (IDA) mode with a mass range of 20–2000 *m*/*z*. All the *N*-glycan species detected were summarized in Table S2 (ESI†).

### 2.2 Data analysis

The data of MALDI-MS and HPLC was processed as the following procedure: *N*-glycans selection, classification and *N*-glycans Analysis (ESI†). For *N*-glycans selection, Kolmogorov–Smirnov test (K–S test) was utilized to reduce the number of features, which was in low correlation with HCC (*p*-value > 0.05). Principal component analysis (PCA), which is usually used as an unsupervised classifier for revealing differences among sample sets, was applied to distinguish HCC cases from healthy controls. The result was then validated by a supervised machine learning method (Table S3 and S4, ESI†). Receiver operating characteristics curve (ROC) is further performed to determine the diagnostic potential for HCC. The area-under-the-curve (AUC) values are considered as highly significant (AUC>0.9), significant (0.8 < AUC<0.9), moderately significant (0.7 < AUC<0.8), less significant (0.6 < AUC<0.7), and insignificant (AUC<0.6).^24–26^ The changes of the significant *N*-glycans for HCC compared with healthy controls were performed with GraphPad Prism 7.0 (GraphPad Software).

It should be noted that the limits of detection (LOD) and quantitation (LOQ) of glycans were measured as the dosages of a standard *N*-glycan of [2-3-0-1-0] giving a signal-to-noise ratio of 3 and 10 respectively, and the detailed parameters were listed in Table S5 (ESI†), and 10 μL of human serum has been used in our study, which met the requirement of detection and quantitation.

## 3. Results and discussion

### 3.1 Selection of individual *N*-glycans

**Fig. 2:**
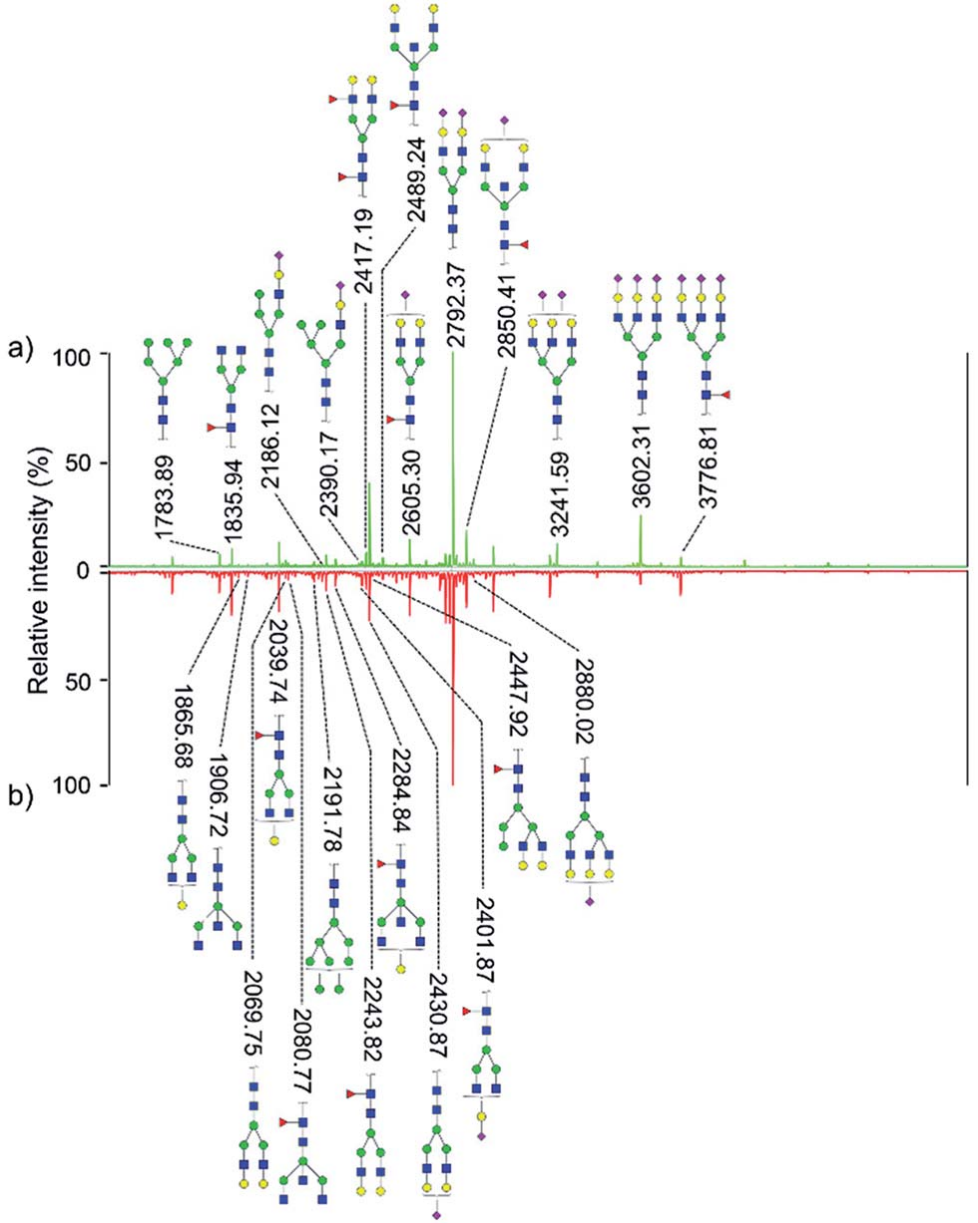
MALDI-MS spectra of permethylated *N*-glycans derived from human serum of healthy controls (a) and HCC cases (b). Symbols: blue squares, HexNAc; green circles, mannose; yellow circles, galactose; red triangles, fucose; purple rhomboid, *N*-acetylneuraminic acid.

**Fig. 3:**
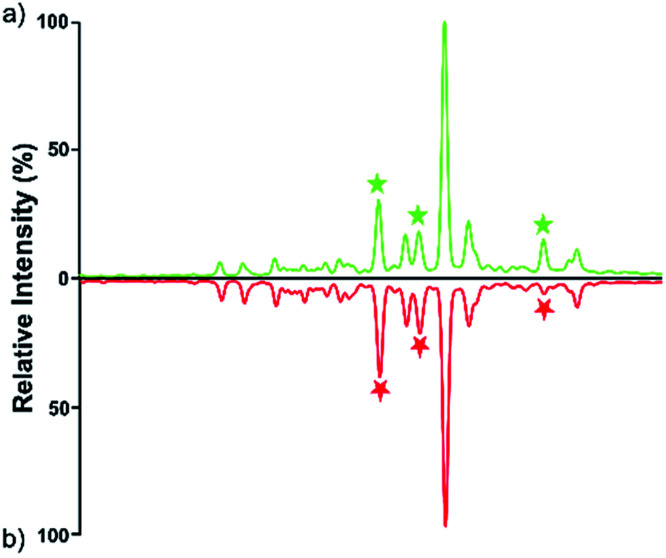
HPLC analysis of 2-AA derivatized *N*-glycans derived from human serum of healthy controls (a) and HCC cases (b).

Quantitative difference in serum glycomic was evaluated between HCC cases and healthy controls (Fig. 2 and 3), and theoretically, an identical set of glycans should be detected and quantified. However, HPLC has presented lower detection sensitivity than MALDI-MS in our study. In addition, more *N*-glycans have been detected by MALDI-MS (Table S1, ESI†). Nevertheless, some *N*-glycans has been detected by MALDI-MS with low abundances, and signal response of these *N*-glycans were too weak, which may cause larger errors in quantitative analysis. Therefore, by a series of data processing such as Peak Deisotoping, Baseline Correction and Noise Filter *etc.*, these glycans which were not suitable for quantitative study have been excluded.

In order to determine which components made more contribution to the differences between sample sets, K–S tests for the normalized peak of *N*-glycans were performed. *N*-glycans with significant difference (*p*-values < 0.05) between HCC cases and healthy controls were listed in Table 1. By MALDI-MS, 19 *N*-glycans have been identified between HCC cases and healthy controls, with *p*-values < 0.05. Meanwhile, 20 specific *N*-glycans were identified with *p*-values < 0.05 by HPLC. It is noteworthy that 10 common *N*-glycans were detected with significant difference whether by MALDI-MS or HPLC. Although the other 9 *N*-glycans detected by MALDI-MS also showed difference, 3 of which showed no difference in HPLC analysis with *p*-values > 0.05, and 6 of which cannot be detected by HPLC. In addition, for the other 10 *N*-glycans detected by HPLC with *p*-values < 0.05, 8 of which showed no difference between HCC cases and healthy controls by MALDI-MS with *p*-values > 0.05, and the signal response of the other 2 *N*-glycans were too weak, which is not suitable for quantitative study.

**Table 1:** AUC value from ROC test, and the average content of *N*-glycans from MALDI-MS and HPLC

*m*/*z*	Composition^a^	Analytical approach	*p*-Value of K–S test	AUC	Average change in HCC^b^
1579.8	[2-5-0-0-0]	HPLC (with [4-3-0-1-0])	3.554 × 10^−7^	0.931	0.816
1661.7	[4-3-0-0-0]	HPLC (with [5-3-0-0-0])	1.006 × 10^−2^	0.793	0.954
1783.9	[2-6-0-0-0]	MALDI-MS	7.291 × 10^−3^	0.756	0.948
1835.8	[4-3-0-1-0]	MALDI-MS	6.308 × 10^−6^	0.897	3.425
		HPLC (with [2-5-0-0-0])	3.554 × 10^−7^	0.931	0.816
1865.9	[4-3-1-0-0]	MALDI-MS	1.741 × 10^−3^	0.739	−0.235
		HPLC (with [5-3-0-1-0])	1.707 × 10^−2^	0.738	0.335
1906.9	[5-3-0-0-0]	HPLC (with [4-3-0-0-0])	1.006 × 10^−2^	0.793	0.954
2040	[4-3-1-1-0]	MALDI-MS	2.496 × 10^−3^	0.847	2.858
2070	[4-3-2-0-0]	MALDI-MS	5.762 × 10^−3^	0.774	−0.495
2081.1	[5-3-0-1-0]	MALDI-MS	5.269 × 10^−4^	0.811	0.641
		HPLC (with [4-3-1-0-0])	1.707 × 10^−2^	0.738	0.335
2186.1	[3-4-1-0-1]	MALDI-MS	1.740 × 10^−3^	0.777	0.291
		HPLC (with [5-3-2-1-0])	6.236 × 10^−4^	0.848	0.447
2192.1	[2-8-0-0-0]	MALDI-MS	2.301 × 10^−5^	0.857	0.732
2244.1	[4-3-2-1-0]	MALDI-MS	5.268 × 10^−4^	0.798	1.156
2285.2	[5-3-1-1-0]	MALDI-MS	3.103 × 10^−6^	0.922	1.179
		HPLC	1.025 × 10^−5^	0.884	0.288
2390.2	[3-5-1-0-1]	MALDI-MS	4.431 × 10^−5^	0.870	0.361
		HPLC (with [4-3-2-1-1])	6.308 × 10^−6^	0.919	1.422
2396.2	[2-9-0-0-0]	MALDI-MS	2.596 × 10^−2^	0.737	0.638
2401.2	[4-3-1-1-1]	MALDI-MS	4.532 × 10^−3^	0.723	−0.385
2417.2	[4-3-2-2-0]	MALDI-MS	3.103 × 10^−6^	0.868	−3.042
2472.2	[5-3-1-0-1]	HPLC (with [5-3-2-2-0])	1.286 × 10^−4^	0.842	0.707
2489.3	[5-3-2-1-0]	MALDI-MS	2.278 × 10^−8^	0.956	0.850
		HPLC (with [3-4-1-0-1])	6.236 × 10^−4^	0.848	0.447
2605.3	[4-3-2-1-1]	MALDI-MS	1.338 × 10^−3^	0.811	1.281
		HPLC (with [3-5-1-0-1])	6.308 × 10^−6^	0.919	1.422
2663.2	[5-3-2-2-0]	HPLC (with [5-3-1-0-1])	1.286 × 10^−4^	0.842	0.707
2676.3	[5-3-2-0-1]	HPLC	1.566 × 10^−2^	0.619	0.019
2792.4	[4-3-2-0-2]	MALDI-MS	1.781 × 10^−2^	0.777	−5.734
		HPLC	6.308 × 10^−6^	0.750	−6.368
2850	[5-3-2-1-1]	HPLC	1.741 × 10^−4^	0.807	−0.950
3054	[5-3-3-1-1]	HPLC	2.933 × 10^−2^	0.677	−0.181
3241.6	[5-3-3-0-2]	MALDI-MS	2.303 × 10^−5^	0.828	−0.883
3211.6	[5-3-2-1-2]	HPLC	4.697 × 10^−2^	0.692	−0.394
3602.8	[5-3-3-0-3]	MALDI-MS	1.566 × 10^−2^	0.770	−0.191
		HPLC	2.863 × 10^−9^	0.976	−3.186
3864.9	[6-3-4-1-2]	HPLC	1.006 × 10^−2^	0.793	−3.784

a The compositions of the *N*-glycans were abbreviated by [*a-b-c-d-e*]: *a* indicates the number of HexNAc, *b* indicates the number of mannose, *c* indicates the number of galactose, *d* indicates the number of fucose and *e* indicates the number of *N*-acetylneuraminic acid.

b The average changes in HCC were calculated by the difference of average intensity of glycans between HCC cases and healthy controls, in which the positive and negative denote the up-regulation and down-regulation respectively.

### 3.2 Principal component analysis and classification of serum *N*-glycan samples

**Fig. 4:**
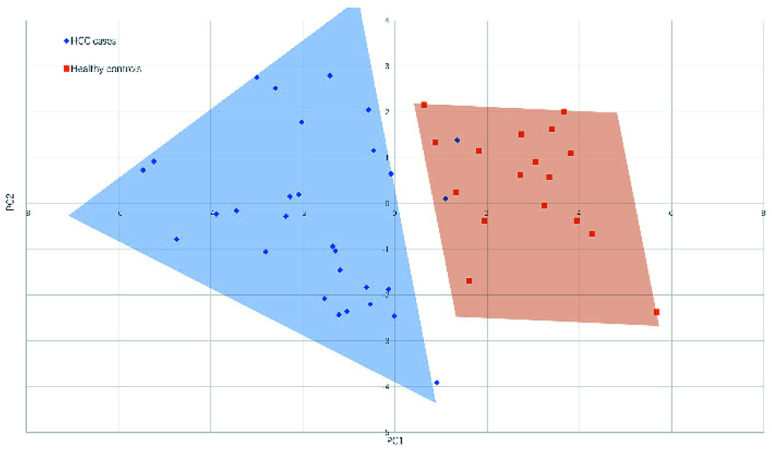
Principal component analysis (PCA) scores plot for HCC cases and healthy controls analyzed by HPLC.

**Fig. 5:**
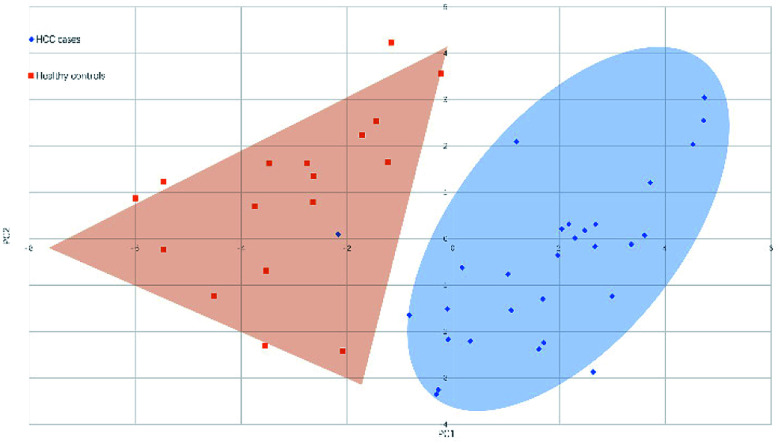
Principal component analysis (PCA) scores plot for HCC cases and healthy controls analyzed by MALDI-MS.

Fig. 4 and 5 illustrated the scores of principal component for the two sample sets analyzed by MALDI-MS and HPLC, respectively. These plots demonstrated that the samples could be obviously distinguished according to the statistically significant changed *N*-glycans, suggesting the capability for the separation of HCC cases from healthy controls by both MALDI-MS and HPLC. Moreover, these results were further verified by classification test with supervised machine learning method. The machine learning method for the data derived from MALDI-MS and HPLC both presented a very good separation between HCC and healthy samples with accuracy more than 90% in classification and cross-validation (Table S3, ESI†). These results suggest that the glycomic changes detected by MALDI-MS and HPLC may be closely associated with HCC. Those *N*-glycans which presented significant difference between HCC and healthy samples in MALDI-MS and HPLC analysis are worthy to be considered as potential biomarker.

### 3.3 Analysis of individual *N*-glycans

In order to evaluate the diagnostic capability of the statistically significant *N*-glycans for HCC, ROC curve analysis was further performed and *N*-glycans with AUC over 0.80 were used in following process.

In our study, 16 *N*-glycans were identified with AUC over 0.80 by MALDI-MS and HPLC. Among which, 5 common *N*-glycans were both identified by these two analytical methods.

**Fig. 6:**
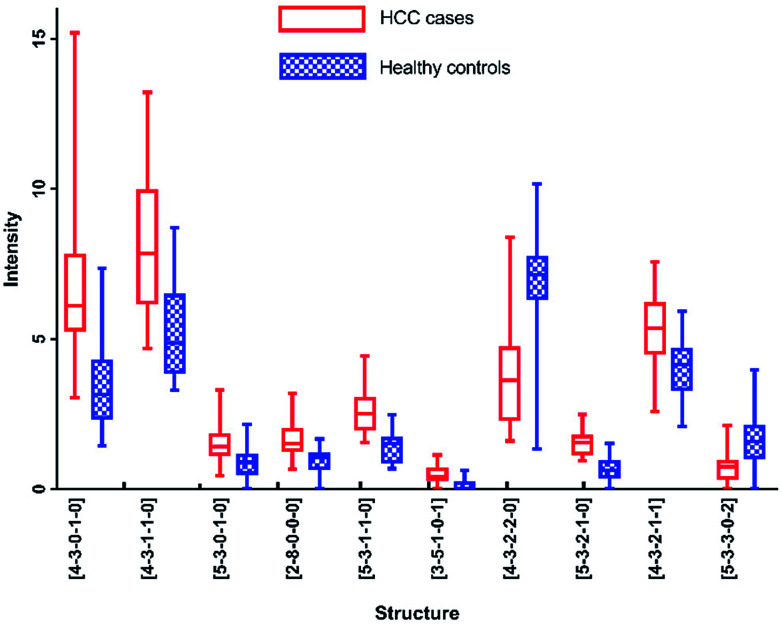
Box-plots of *N*-glycans with AUC over 0.8 in MALDI-MS analysis.

As listed in Table 1, *N*-glycans analyzed by MALDI-MS with AUC over 0.80 were as following: [4-3-0-1-0], [4-3-1-1-0], [5-3-0-1-0], [2-8-0-0-0], [5-3-1-1-0], [3-5-1-0-1], [4-3-2-2-0], [5-3-2-1-0], [4-3-2-1-1] and [5-3-3-0-2]. Fig. 6 presented these significantly changed *N*-glycans, of which 8 were up-regulated, especially for glycans with fucosylated moieties, and that 2 glycans were down-regulated in patients with HCC.

**Fig. 7:**
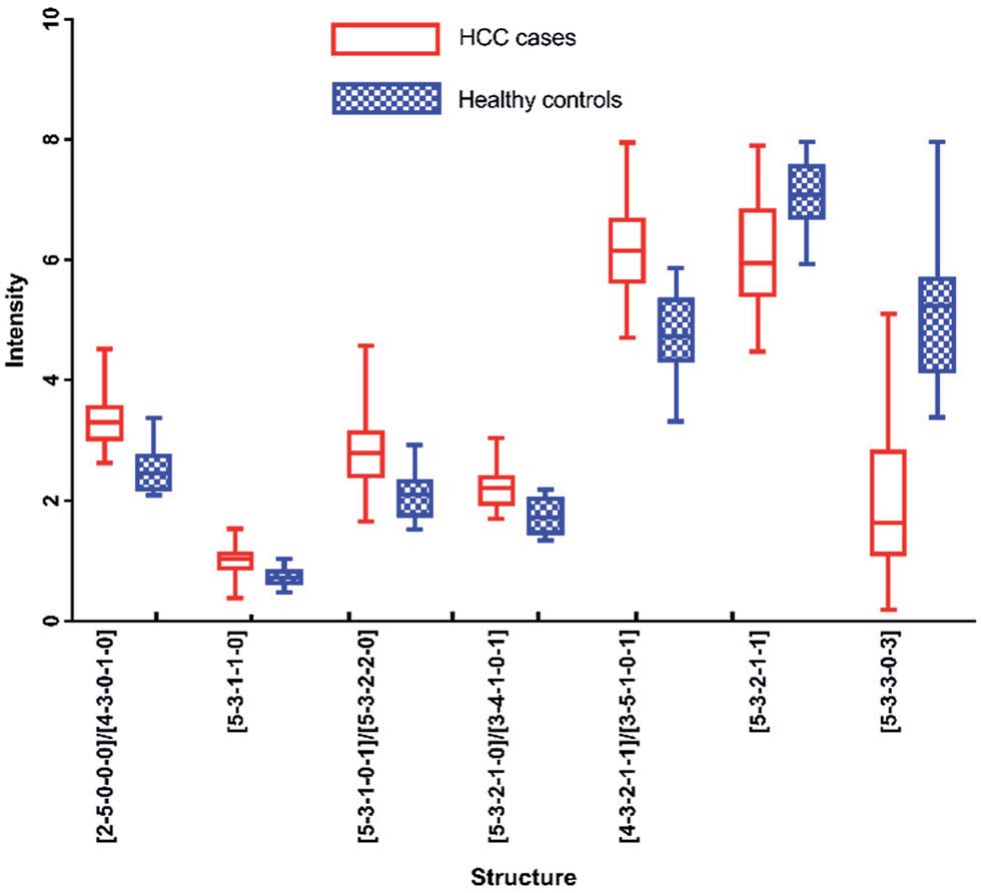
Box-plots of *N*-glycans with AUC over 0.8 in HPLC analysis.

In addition, *N*-glycans with AUC over 0.80 have been identified by HPLC, including [5-3-3-0-3], [2-5-0-0-0]/[4-3-0-1-0], [4-3-2-1-1]/[3-5-1-0-1], [5-3-1-1-0], [5-3-2-1-1], [5-3-2-1-0]/[3-4-1-0-1] and [5-3-1-0-1]/[5-3-2-2-0]. The changes of these peaks for HCC compared to healthy controls were shown in Fig. 7, of which 5 were up-regulated and 2 were down-regulated. It also should be noted that four of these peaks contained co-elution with two glycan structures. And the comparison of the results analyzed by these two analytical methods will be performed in following process.

#### 3.3.1 The common significantly altered *N*-glycans identified by MALDI-MS and HPLC

5 common *N*-glycans with significance (AUC over 0.8) have been identified by MALDI-MS and HPLC, including [4-3-0-1-0], [5-3-1-1-0], [3-5-1-0-1], [5-3-2-1-0] and [4-3-2-1-1]. As shown in Table 1, all these five *N*-glycans were up-regulated, of which [4-3-0-1-0], [5-3-1-1-0], [5-3-2-1-0] and [4-3-2-1-1] are complex glycans with bi-antennary structure and [3-5-1-0-1] is hybrid glycan. Moreover, four of these *N*-glycans are fucosylated, indicating that fucosylation may be related to HCC, which is consistent with a previous report.^27^

Furthermore, each of these *N*-glycans presented highly significant level (AUC over 0.9) in one analytical method, however showed significant level (0.8 < AUC < 0.9) in another method. For example, [5-3-1-1-0] reflected the optimal diagnostic capability for distinguishing HCC cases from healthy controls with AUC of 0.922 by MALDI-MS, while with AUC of 0.884 by HPLC. The results described above showed that these five *N*-glycans were all highly correlated with HCC. More interestingly, [5-3-2-1-0] and [5-3-1-1-0] have been reported to be potential biomarkers for HCC by some previous studies,^12,13,28^ while [4-3-0-1-0], [3-5-1-0-1] and [4-3-2-1-1] haven't been reported, suggesting that these *N*-glycans may be the potential biomarkers for HCC.

#### 3.3.2 The significantly altered *N*-glycans only identified by MALDI-MS

[4-3-1-1-0], [5-3-0-1-0], [2-8-0-0-0], [4-3-2-2-0] and [5-3-3-0-2] are significant (AUC over 0.8) only by MALDI-MS analysis. 3 of these *N*-glycans are up-regulated, and 2 of which are down-regulated. In addition, [4-3-1-1-0], [5-3-0-1-0] and [4-3-2-2-0] are bi-antennary, [5-3-3-0-2] is tri-antennary, and [2-8-0-0-0] is high mannose glycan. Moreover, [4-3-1-1-0] and [5-3-3-0-2] have been reported to endure an up-regulation in previous studies,^12,13^ which is almost consistent with our study.

Although these 5 *N*-glycans presented significant difference between HCC cases and healthy controls by MALDI-MS, they showed low significant levels in HPLC analysis. For example, [4-3-1-1-0] and [5-3-3-0-2] showed no significance with *p*-value > 0.05. Additionally, [2-8-0-0-0] and [4-3-2-2-0] have not been detected by HPLC analysis. It is noteworthy that [5-3-0-1-0] and [4-3-1-0-0] are involved in the same chromatographic peak with moderately significant difference with AUC of 0.738 between HCC cases and healthy controls. However, [5-3-0-1-0] showed significant difference with AUC of 0.811 in the analysis of MALDI-MS, which is higher than that in HPLC analysis. The lower significant difference of the chromatographic peak containing [5-3-0-1-0] and [4-3-1-0-0] may be caused by different regulation trends of these two glycans, of which [4-3-1-0-0] has been identified with down-regulation in MALDI-MS, while [5-3-0-1-0] presented up-regulation in MALDI-MS, the difference of the chromatographic peak has been reduced by these two glycans, suggesting the complementarity of these two analytical methods in analysis of serum glycomics.

#### 3.3.3 The significantly altered *N*-glycans only identified by HPLC

[5-3-1-0-1]/[5-3-2-2-0], [5-3-2-1-1], [2-5-0-0-0], [3-4-1-0-1] and [5-3-3-0-3] are significant (AUC over 0.8) only by HPLC analysis. 4 of these glycans are up-regulated, and 2 of them are down-regulated. [5-3-1-0-1] and [5-3-2-2-0] were co-eluted in the same chromatographic peak, [2-5-0-0-0] and [4-3-0-1-0] were co-eluted, meanwhile [3-4-1-0-1] and [5-3-2-1-0] were also co-eluted. [5-3-1-0-1], [5-3-2-2-0] and [5-3-2-1-1] are bi-antennary, [5-3-3-0-3] is tri-antennary, [2-5-0-0-0] is high mannose glycan, [3-4-1-0-1] is hybrid glycan, and 2 of the 6 *N*-glycans are fucosylated. In addition, [5-3-3-0-3] has been reported to endure a down-regulation in previous study,^12^ which is almost consistent with our result.

Although these 6 *N*-glycans showed significant difference by HPLC, they showed lower significant levels in MALDI-MS analysis. For example, there is no significant difference between HCC cases and healthy controls for [5-3-1-0-1]/[5-3-2-2-0], [2-5-0-0-0] and [5-3-2-1-1] by MALDI-MS. Meanwhile, [3-4-1-0-1] and [5-3-3-0-3] presented moderate significance (0.7 < AUC<0.8) between HCC cases and healthy controls by MALDI-MS, which were lower than HPLC analysis.

In addition, it should be noted that [3-4-1-0-1] and [5-3-2-1-0] were co-eluted in the same chromatographic peak with significant difference (AUC of 0.848) by HPLC. However, [3-4-1-0-1] showed moderate significance with AUC of 0.777 and [5-3-2-1-0] presented high significance with AUC of 0.956 by MALDI-MS. The significant difference of the co-eluted chromatographic peak in HPLC may be caused by the mixing of [3-4-1-0-1] and [5-3-2-1-0], suggesting these two analytical methods were complementary in potential biomarker discovery for HCC.

### 3.4 Difference of MALDI-MS and HPLC in identification of biomarkers

As described above, MALDI-MS and HPLC have identified 16 *N*-glycans significantly related with HCC (AUC > 0.8), 5 common *N*-glycans were detected with significant difference whether by MALDI-MS or HPLC, 11 *N*-glycans are only detected with AUC over 0.8 by only one analytical approach, 5 are identified only by MALDI-MS, 6 are identified only by HPLC.

It should be noted that among these 11 *N*-glycans, almost half (5/11) were co-eluted with other *N*-glycans. In addition, a previous study has reported that the co-elution of chromatographic peaks in HPLC analysis might limit the quantitation of *N*-glycans.^29^ In our study, the separation capability of HPLC is limited for analysing *N*-glycans with bisecting structures, which may interfere the identification of biomarker, and may be one of the major causes that lead to the differences of glycomics between HPLC and MALDI-MS analysis, and the co-eluted glycans associated with the detection limitation need to be further validated. Interestingly, MALDI-MS analysis in our study present capability of validation the effectiveness of co-eluted *N*-glycans in HPLC. For example, [5-3-0-1-0] performs significant statistical difference between HCC cases and healthy controls by MALDI-MS, while it showed lower significance by HPLC analysis, which might be interfered by [4-3-1-0-0] in the co-eluting chromatographic peak. In addition, the chromatographic peak containing [2-5-0-0-0] and [4-3-0-1-0] is identified with high significance by HPLC analysis, meanwhile [4-3-0-1-0] showed significant difference by MALDI-MS, while [2-5-0-0-0] presented no significance between HCC cases and healthy controls by MALDI-MS, suggesting the significant difference of the chromatographic peak containing [2-5-0-0-0] and [4-3-0-1-0] may be caused by [4-3-0-1-0], further indicated the complementarity of these two analytical methods in analysis of serum glycomics.

Meanwhile, 2 of these 11 are tri-antennary *N*-glycans. [5-3-3-0-2] has been identified as significant by MALDI-MS, while [5-3-3-0-3] has been identified by HPLC. Wada *etc.* had expressed that the levels of some tri-antennary glycans determined by MALDI-MS were also different from chromatographic analysis, but it is not possible to decide which approach is more precise.^30^ As described above, both these 2 tri-antennary *N*-glycans have been reported in previous studies,^12,13^ indicating that the tri-antennary as potential biomarker could be promising whether it identified by MALDI-MS or HPLC, and the complementarity of these two analytical methods in analysis of serum glycomics.

Certainly, besides the causes described above, further studies for other factors which may lead to the differences of glycomics between HPLC and MALDI-MS analysis is indeed required. Interestingly, the deep study for the differences may help expanding the biomarker library for HCC to a certain extent. For example, [4-3-1-1-0] has been only identified by MALDI-MS with AUC over 0.8, which was related to HCC in a previous study.^12^

Additionally, differences also existed between MALDI-MS and HPLC in terms of these analytical platforms. MALDI-MS presented excellent reproducibility, high throughput and low consumption of samples.^30^ While, HPLC showed precise intensities and reproducibility in detection of *N*-glycans.^31^ Overall, all these results described above indicated that these two analytical methods are complementary for identifying biomarkers of HCC.

## 4. Conclusions

In this study, a strategy coupled with machine learning is firstly applied for comparing the analytical results of MALDI-MS and HPLC on the same serum glycomics of HCC samples, reducing various physiological and environmental factors which may cause the aberrant changes in glycosylation. The identification capability for HCC samples based on MALDI-MS and HPLC were validated by a supervised machine learning method. In addition, by these two analytical methods, some specific *N*-glycans which maybe the potential biomarkers for HCC have been identified respectively, which expanded the biomarker library for HCC analysis. Furthermore, the similarities and differences in *N*-glycans analysis by MALDI-MS and HPLC have also been investigated. Overall, all these results above demonstrated that these two analytical methods are complementary in analysis of *N*-glycans which were highly correlated with HCC. Meanwhile, it also should be noted that this work is just a preliminary step toward the discovery of potential biomarkers for HCC and further perspective studies with larger sample sets is indeed required before the clinical application. Additionally, the comparison of MALDI-MS and HPLC in glycomic analysis might offer an effective platform for identifying biomarkers for other cancers studies in the future.

## Conflicts of interest

There are no conflicts to declare.

## Supplementary Material

RA-008-C8RA02542H-s001
