# Supplementary material for: Comparison of the methods for profiling N-glycans—hepatocellular carcinoma serum glycomics study
Source: RSC Adv. 2018 Jul 20;8(46):26116–23. doi: 10.1039/c8ra02542h (PMC9082735; doi:10.1039/c8ra02542h)
Supplement: RA-008-C8RA02542H-s001 [file RA-008-C8RA02542H-s001.pdf]

Electronic Supplementary Material (ESI) for RSC Advances.
This journal is © The Royal Society of Chemistry 2018

# SUPPORTING INFORMATION

# Comparison of the methods for profiling N-glycans—Hepatocellular carcinoma serum glycomics study

**Ran Wang[a†], Yufei Liu[a†], Chang Wang[a†], Henghui Li[a], Xin Liu[a*], Liming Cheng[b*] and Yanhong Zhou[a*]**

***a Britton Chance Center for Biomedical Photonics at Wuhan National Laboratory for Optoelectronics – Hubei Bioinformatics & Molecular Imaging Key Laboratory, Systems Biology Theme, Department of Biomedical Engineering, College of Life Science and Technology, Huazhong University of Science and Technology, Wuhan 430074, China***

***b Department of Laboratory Medicine, Tongji Hospital, Wuhan 430074, China***

†These authors contributed equally to this work.

*Corresponding authors:

Dr. Yanhong Zhou, Department of Biomedical Engineering, College of Life Science and Technology, Huazhong University of Science and Technology, Wuhan 430074, China

Email: yhzhou@hust.edu.cn, Tel: +86-27-87792217

Dr. Liming Cheng, Department of Laboratory Medicine, Tongji Hospital, Wuhan 430074, China

Email:chengliming2002@163.com

Dr. Xin Liu, Department of Biomedical Engineering, College of Life Science and Technology, Huazhong University of Science and Technology, Wuhan 430074, China

Email: xliu@mail.hust.edu.cn, Tel: +86-27-87793180

# Method Section

## Classification

Supervised machine learning method was performed for classification of healthy versus HCC whole serum samples. The method employs principal components analysis (PCA) followed by linear discriminant analysis (LDA) on MALDI-MS and HPLC data. First, PCA is performed for the reduction of the dimensionality of the feature space to avoid overfitting, and LDA is then performed in the low-dimensional space to classify the sample points.

PCA was used to transfer the space of sample points into a lower dimensional space with little information loss. By computing covariance matrix of the sample points, the eigenvectors with large eigenvalue determined the principal components. Most sample point variance is presented by the first few principal components with largest eigenvalues.

LDA computes the discriminant to classify the sample points by maximizing the between-class scatter and minimizing the within-class scatter. The discriminant can be used to predict the class membership of an unknown sample.

## Cross-validation

Cross-validation is used to test the predict power of supervised machine learning method. This paper preferred LOOCV to verify the accuracy and the predictive power of the PCA-LDA approach (Table S3). This was repeated such that each sample in the dataset was used once as the validation data. When a sample was validation data, it had not been seen by the method, either PCA or LDA before, and its classifications were also unknown.

## Comparison with other classifier

In order to indicate the advantages of LDA in MALDI-MS and HPLC data classification, Naïve Bayes (NB) classifier and Random Forest (RF) classifier are utilized to separate the same experimental data, respectively. And the leave one out cross validation (LOOCV) is employed for cross-verification. By comparing the sensitivity and the specificity of these three classification methods for MALDI-MS data and HPLC data, in Table S4, it shows the effectiveness of these classifiers. It indicates that the data derived from MALDI-MS and HPLC both contained important information for N-glycans associated with HCC.

## Results and discussion section

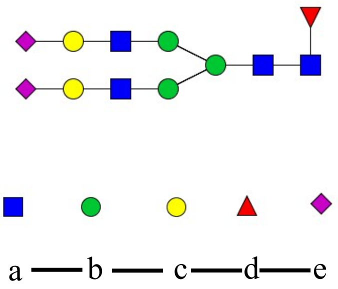


Scheme 1. The compositions of the N-glycans were abbreviated by [a-b-c-d-e]: a indicates the number of HexNAc, b indicates the number of mannose, c indicates the number of galactose, d indicates the number of fucose and e indicates the number of N-acetylneuraminic acid. An example of glycan structure nomenclature was used in our study. The model glycan structure contains 4 HexNAc, 3 Mannose, 2 Galactose, 1 Fucose and 2 Neuraminic acid residues. Namely, the nomenclature is [4-3-2-1-2].

**Table S1** Permethylated N-glycans released from human serum identified by MALDI-MS.

| No. | m/z | N-glycan structure | C.Composition | M.Composition |
|---|---|---|---|---|
| 1 | 1579.74 | 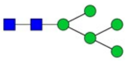 | $Hex_5HexNAc_2$ | [2-5-0-0-0] |
| 2 | 1620.81 | 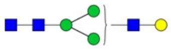 | $Hex_4HexNAc_3$ | [3-3-1-0-0] |
| 3 | 1661.82 | 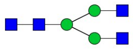 | $Hex_3HexNAc_4$ | [4-3-0-0-0] |
| 4 | 1783.83 | 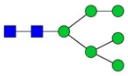 | $Hex_6HexNAc_2$ | [2-6-0-0-0] |
| 5 | 1824.89 | 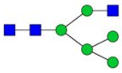 | $Hex_5HexNAc_3$ | [3-5-0-0-0] |
| 6 | 1835.87 | 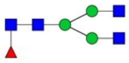 | $Hex_3HexNAc_4DeoxyHex_1$ | [4-3-0-1-0] |
| 7 | 1865.98 | 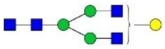 | $Hex_4HexNAc_4$ | [4-3-1-0-0] |
| 8 | 1906.92 | 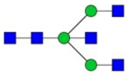 | $Hex_3HexNAc_5$ | [5-3-0-0-0] |
| 9 | 1987.89 | 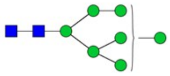 | $Hex_7HexNAc_2$ | [2-7-0-0-0] |
| 10 | 1982.02 | 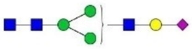 | $Hex_4HexNAc_3Neu5Ac_1$ | [3-3-1-0-1] |
| 11 | 1998.99 | 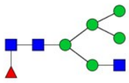 | $Hex_5HexNAc_3DeoxyHex_1$ | [3-5-0-1-0] |
| 12 | 2028.95 | 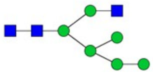 | $Hex_6HexNAc_3$ | [3-6-0-0-0] |
| 13 | 2039.95 | 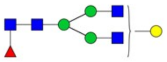 | $Hex_4HexNAc_4DeoxyHex_1$ | [4-3-1-1-0] |

| | | | | |
|---|---|---|---|---|
| 14 | 2070.08 | 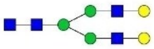 | $Hex_5HexNAc_4$ | [4-3-2-0-0] |
| 15 | 2081.08 | 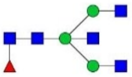 | $Hex_3HexNAc_5DeoxyHex_1$ | [5-3-0-1-0] |
| 16 | 2111.08 | 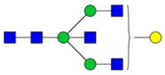 | $Hex_4HexNAc_5$ | [5-3-1-0-0] |
| 17 | 2156.12 | 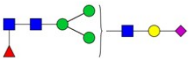 | $Hex_4HexNAc_3Neu5Ac_1DeoxyHex_1$ | [3-3-1-1-1] |
| 18 | 2186.13 | 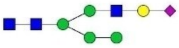 | $Hex_5HexNAc_3Neu5Ac_1$ | [3-4-1-0-1] |
| 19 | 2192.00 | 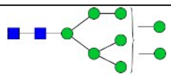 | $Hex_8HexNAc_2$ | [2-8-0-0-0] |
| 20 | 2227.15 | 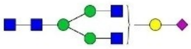 | $Hex_4HexNAc_4Neu5Ac_1$ | [4-3-1-0-1] |
| 21 | 2244.04 | 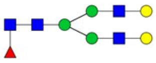 | $Hex_5HexNAc_4DeoxyHex_1$ | [4-3-2-1-0] |
| 22 | 2285.19 | 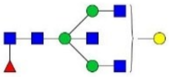 | $Hex_4HexNAc_5DeoxyHex_1$ | [5-3-1-1-0] |
| 23 | 2315.20 | 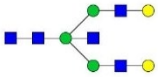 | $Hex_5HexNAc_5$ | [5-3-2-0-0] |
| 24 | 2390.23 | 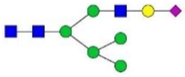 | $Hex_6HexNAc_3Neu5Ac_1$ | [3-5-1-0-1] |
| 25 | 2396.22 | 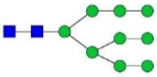 | $Hex_9HexNAc_2$ | [2-9-0-0-0] |
| 26 | 2401 | 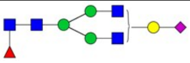 | $Hex_4HexNAc_4Neu5Ac_1DeoxyHex_1$ | [4-3-1-1-1] |
| 27 | 2417.21 | 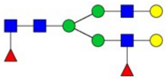 | $Hex_5HexNAc_4DeoxyHex_2$ | [4-3-2-2-0] |
| 28 | 2431.12 | 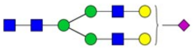 | $Hex_5HexNAc_4Neu5Ac_1$ | [4-3-2-0-1] |

| | | | | |
|---|---|---|---|---|
| 29 | 2448.22 | 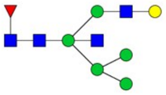 | $Hex_6HexNAc_4DeoxyHex_1$ | [4-5-1-1-0] |
| 30 | 2472.26 | 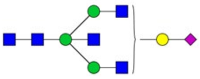 | $Hex_4HexNAc_5Neu5Ac_1$ | [5-3-1-0-1] |
| 31 | 2489.15 | 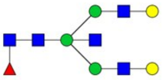 | $Hex_5HexNAc_5DeoxyHex_1$ | [5-3-2-1-0] |
| 32 | 2519.26 | 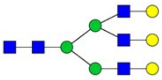 | $Hex_6HexNAc_5$ | [5-3-3-0-0] |
| 33 | 2592.30 | 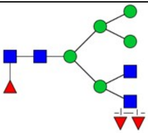 | $Hex_5HexNAc_4DeoxyHex_3$ | [4-5-0-3-0] |
| 34 | 2605.19 | 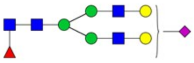 | $Hex_5HexNAc_4Neu5Ac_1DeoxyHex_1$ | [4-3-2-1-1] |
| 35 | 2622.31 | 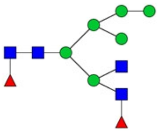 | $Hex_6HexNAc_4DeoxyHex_2$ | [4-6-0-2-0] |
| 36 | 2635.30 | 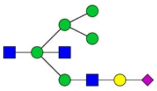 | $Hex_6HexNAc_3Neu5Ac_1$ | [3-5-1-0-1] |
| 37 | 2646.37 | 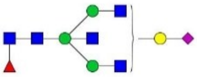 | $Hex_4HexNAc_5Neu5Ac_1DeoxyHex_1$ | [5-3-1-1-1] |
| 38 | 2652.32 | 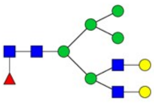 | $Hex_7HexNAc_4DeoxyHex_1$ | [4-5-2-1-0] |
| 39 | 2663.34 | 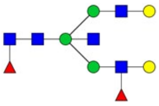 | $Hex_5HexNAc_5DeoxyHex_2$ | [5-3-2-2-0] |

| | | | | |
|---|---|---|---|---|
| 40 | 2676.26 | 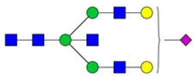 | $Hex_5HexNAc_5Neu5Ac_1$ | [5-3-2-0-1] |
| 41 | 2693.35 | 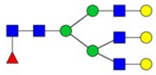 | $Hex_6HexNAc_5DeoxyHex_1$ | [5-3-3-1-0] |
| 42 | 2792.28 | 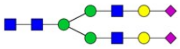 | $Hex_5HexNAc_4Neu5Ac_2$ | [4-3-2-0-2] |
| 43 | 2809.39 | 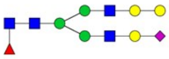 | $Hex_6HexNAc_4Neu5Ac_1DeoxyHex_1$ | [4-3-3-1-1] |
| 44 | 2850.31 | 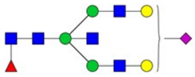 | $Hex_5HexNAc_5Neu5Ac_1DeoxyHex_1$ | [5-3-2-1-1] |
| 45 | 2880.42 | 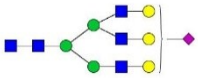 | $Hex_6HexNAc_5Neu5Ac_1$ | [5-3-3-0-1] |
| 46 | 2925.46 | 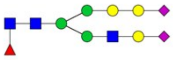 | $Hex_6HexNAc_3Neu5Ac_2DeoxyHex_1$ | [3-3-3-1-2] |
| 47 | 2966.36 | 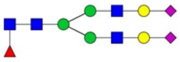 | $Hex_5HexNAc_4Neu5Ac_2DeoxyHex_1$ | [4-3-2-1-2] |
| 48 | 3013.49 | 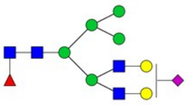 | $Hex_7HexNAc_4Neu5Ac_1DeoxyHex_1$ | [4-5-2-1-1] |
| 49 | 3037.51 | 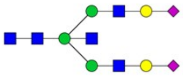 | $Hex_5HexNAc_5Neu5Ac_2$ | [5-3-2-0-2] |
| 50 | 3054.38 | 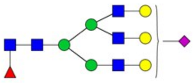 | $Hex_6HexNAc_5Neu5Ac_1DeoxyHex_1$ | [5-3-3-1-1] |

| | | | | |
|---|---|---|---|---|
| 51 | 3142.57 | 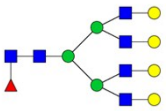 | $Hex_7HexNAc_6DeoxyHex_1$ | [6-3-4-1-0] |
| 52 | 3211.47 | 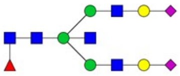 | $Hex_5HexNAc_5Neu5Ac_2DeoxyHex_1$ | [5-3-2-1-2] |
| 53 | 3228.61 | 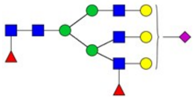 | $Hex_6HexNAc_5Neu5Ac_1DeoxyHex_2$ | [5-3-3-2-1] |
| 54 | 3241.46 | 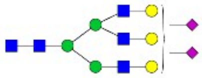 | $Hex_6HexNAc_5Neu5Ac_2$ | [5-3-3-0-2] |
| 55 | 3269.64 | 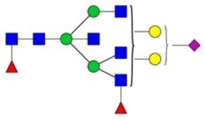 | $Hex_5HexNAc_6Neu5Ac_1DeoxyHex_2$ | [6-3-2-2-1] |
| 56 | 3329.66 | 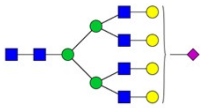 | $Hex_7HexNAc_6Neu5Ac_1$ | [6-3-4-0-1] |
| 57 | 3385.69 | 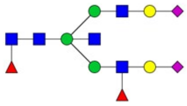 | $Hex_5HexNAc_5Neu5Ac_2DeoxyHex_2$ | [5-3-2-2-2] |
| 58 | 3402.70 | 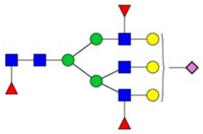 | $Hex_6HexNAc_5Neu5Ac_1DeoxyHex_3$ | [5-3-3-3-1] |
| 59 | 3415.54 | 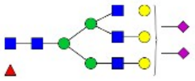 | $Hex_6HexNAc_5Neu5Ac_2DeoxyHex_1$ | [5-3-3-1-2] |
| 60 | 3602.64 | 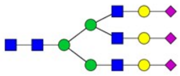 | $Hex_6HexNAc_5Neu5Ac_3$ | [5-3-3-0-3] |
| 61 | 3690.79 | 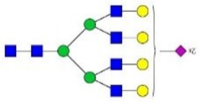 | $Hex_7HexNAc_6Neu5Ac_2$ | [6-3-4-0-2] |

| | | | | |
|---|---|---|---|---|
| 62 | 3776.72 | 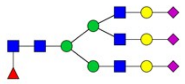 | Hex$_6$HexNAc$_5$Neu5Ac$_3$DeoxyHex$_1$ | [5-3-3-1-3] |
| 63 | 3864.92 | 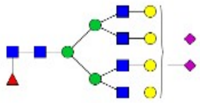 | Hex$_7$HexNAc$_6$Neu5Ac$_2$DeoxyHex$_1$ | [6-3-4-1-2] |
| 64 | 3950.96 | 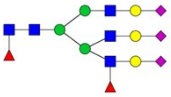 | Hex$_6$HexNAc$_5$Neu5Ac$_3$DeoxyHex$_2$ | [5-3-3-2-3] |
| 65 | 3963.95 | 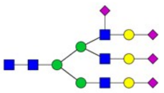 | Hex$_6$HexNAc$_5$Neu5Ac$_4$ | [5-3-3-0-4] |
| 66 | 4051.87 | 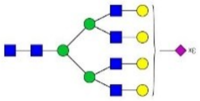 | Hex$_7$HexNAc$_6$Neu5Ac$_3$ | [6-3-4-0-3] |
| 67 | 4225.89 | 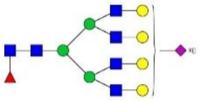 | Hex$_7$HexNAc$_6$Neu5Ac$_3$DeoxyHex$_1$ | [6-3-4-1-3] |
| 68 | 4413.03 | 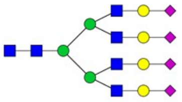 | Hex$_7$HexNAc$_6$Neu5Ac$_4$ | [6-3-4-0-4] |
| 69 | 4587.18 | 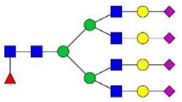 | Hex$_7$HexNAc$_6$Neu5Ac$_4$DeoxyHex$_1$ | [6-3-4-1-4] |
| 70 | 4761.29 | 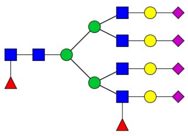 | Hex$_7$HexNAc$_6$Neu5Ac$_4$DeoxyHex$_2$ | [6-3-4-2-4] |

Abbreviations: hexose (Hex), N-acetylhexosamine (HexNAc), N-acetylneuraminic acid (Neu5Ac) and fucose (DeoxyHex). All peaks detected by MALDI were single sodium adducts.

**Table S2** The N-glycans released from human serum sample by HPLC.

| Peak No. | m/z | N-glycan structure | C.Composition | M.Composition |
|---|---|---|---|---|
| 1 | 691.26 | 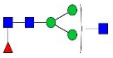 | $Hex_3HexNAc_3DeoxyHex_1$ | [3-3-0-1-0] |
| 2 | 719.77 |  | $Hex_3HexNAc_4$ | [4-3-0-0-0] |
| | 821.31 | 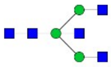 | $Hex_3HexNAc_5$ | [5-3-0-0-0] |
| 3 | 678.75 | 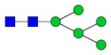 | $Hex_5HexNAc_2$ | [2-5-0-0-0] |
| | 792.80 | 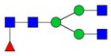 | $Hex_3HexNAc_4DeoxyHex_1$ | [4-3-0-1-0] |
| 4 | 894.34 | 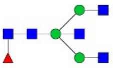 | $Hex_3HexNAc_5DeoxyHex_1$ | [5-3-0-1-0] |
| | 800.80 | 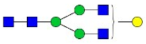 | $Hex_4HexNAc_4$ | [4-3-1-0-0] |
| 5 | 902.34 | 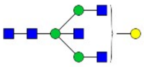 | $Hex_4HexNAc_5$ | [5-3-1-0-0] |
| 6 | 873.83 | 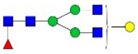 | $Hex_4HexNAc_4DeoxyHex_1$ | [4-3-1-1-0] |
| 7 | 975.37 | 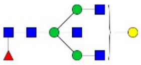 | $Hex_4HexNAc_5DeoxyHex_1$ | [5-3-1-1-0] |
| 8 | 844.81 | 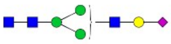 | $Hex_4HexNAc_3Neu5Ac_1$ | [3-3-1-0-1] |
| 9 | 917.84 | 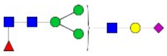 | $Hex_3HexNAc_3Neu5Ac_1DeoxyHex_1$ | [3-3-1-1-1] |
| 10 | 946.35 | 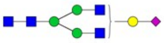 | $Hex_4HexNAc_4Neu5Ac_1$ | [4-3-1-0-1] |

| | | | | |
|---|---|---|---|---|
| 11 | 1047.89 | 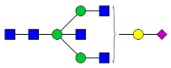 | $Hex_4HexNAc_5Neu5Ac_1$ | [5-3-1-0-1] |
| | 1129.42 | 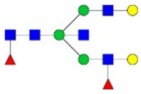 | $Hex_5HexNAc_5DeoxyHex_2$ | [5-3-2-2-0] |
| 12 | 1056.39 | 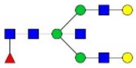 | $Hex_5HexNAc_5DeoxyHex_1$ | [5-3-1-0-1] |
| | 925.83 | 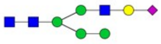 | $Hex_5HexNAc_3Neu5Ac_1$ | [5-3-2-2-0] |
| 13 | 1019.38 | 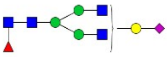 | $Hex_4HexNAc_4Neu5Ac_1DeoxyHex_1$ | [4-3-1-1-1] |
| 14 | 1120.91 | 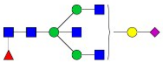 | $Hex_4HexNAc_5Neu5Ac_1DeoxyHex_1$ | [5-3-1-1-1] |
| 15 | 1027.37 | 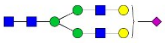 | $Hex_5HexNAc_4Neu5Ac_1$ | [4-3-2-0-1] |
| 16 | 1128.91 | 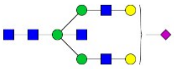 | $Hex_5HexNAc_5Neu5Ac_1$ | [5-3-2-0-1] |
| 17 | 1100.40 | 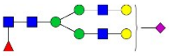 | $Hex_5HexNAc_4Neu5Ac_1DeoxyHex_1$ | [4-3-2-1-1] |
| | 1006.86 | 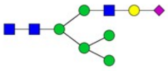 | $Hex_6HexNAc_3Neu5Ac_1$ | [3-5-1-0-1] |
| 18 | 1201.91 | 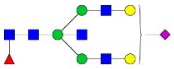 | $Hex_5HexNAc_5Neu5Ac_1DeoxyHex_1$ | [5-3-2-1-1] |
| 19 | 1209.94 | 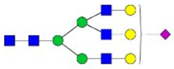 | $Hex_6HexNAc_5Neu5Ac_1$ | [5-3-3-0-1] |
| 20 | 1172.92 | 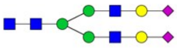 | $Hex_5HexNAc_4Neu5Ac_2$ | [4-3-2-0-2] |
| 21 | 1274.46 |  | $Hex_5HexNAc_5Neu5Ac_2$ | [5-3-2-0-2] |
| 22 | 1245.95 | 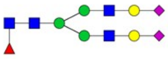 | $Hex_5HexNAc_4Neu5Ac_2DeoxyHex_1$ | [4-3-2-1-2] |
| 23 | 1347.49 | 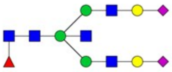 | $Hex_5HexNAc_5Neu5Ac_2DeoxyHex_1$ | [5-3-2-1-2] |
| 24 | 1282.97 | 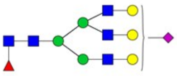 | $Hex_6HexNAc_5Neu5Ac_1DeoxyHex_1$ | [5-3-3-1-1] |
| 25 | 1355.49 |  | $Hex_6HexNAc_5Neu5Ac_2$ | [5-3-3-0-2] |

| | | | | |
|---|---|---|---|---|
| 26 | 1428.52 |  | $Hex_6HexNAc_5Neu5Ac_2DeoxyHex_1$ | [5-3-3-1-2] |
| 27 | 1356.49 | 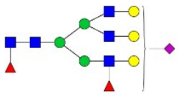 | $Hex_6HexNAc_5Neu5Ac_1DeoxyHex_2$ | [5-3-3-2-1] |
| 28 | 1465.53 | 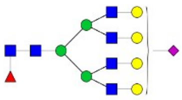 | $Hex_7HexNAc_6Neu5Ac_1DeoxyHex_1$ | [6-3-4-1-1] |
| 29 | 1501.03 | 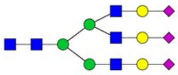 | $Hex_6HexNAc_5Neu5Ac_3$ | [5-3-3-0-3] |
| 30 | 1611.08 |  | $Hex_7HexNAc_6Neu5Ac_2DeoxyHex_1$ | [6-3-4-1-2] |
| 31 | 1574.06 | 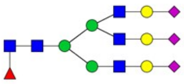 | $Hex_6HexNAc_5Neu5Ac_3DeoxyHex_1$ | [5-3-3-1-3] |

Abbreviations: hexose (Hex), N-acetylhexosamine (HexNAc), N-acetylneuraminic acid (Neu5Ac) and fucose (DeoxyHex). In order to confirm the chemical compositions of 2-AA derivatized N-glycans, the collections of each peak from HPLC were further analyzed by nanoLC-ESI-MS.

**Table S3** Sensitivity, specificity, positive and negative predictive power of the LDA method.

| | | Sensitivity | specificity | positive predictive | negative predictive |
|---|---|---|---|---|---|
| MALDI-MS | Classified groups | | | | |
| | HCC vs. control | 96.55 | 100 | 96.55 | 100 |
| | Predicted groups (cross-validation) | | | | |
| | HCC vs. control | 96.55 | 96.55 | 96.55 | 94.11 |
| HPLC | Classified groups | | | | |
| | HCC vs. control | 93.10 | 100 | 93.10 | 100 |
| | Predicted groups (cross-validation) | | | | |
| | HCC vs. control | 93.10 | 96.42 | 93.10 | 94.11 |

**Table S4** Sensitivity of LDA, NB and RF classifier for MALDI-MS and HPLC data.

| Data | Indicator | LDA | NB | RF |
|---|---|---|---|---|
| MALDI-MS | Accuracy | 0.9655 | 0.8913 | 0.9565 |

| | | | | |
|---|---|---|---|---|
| HPLC | | 0.9310 | 0.9574 | 0.9130 |

**Table S5** The LOD and LOQ values for standard N-glycan of [2-3-0-1-0] by HPLC and MALDI-MS analysis

| Parameters / Standard glycan / Methods | HPLC | MALDI-MS |
|---|---|---|
| LOD (3) | 0.176 μM (Load volume: 5μL) | 9.48 nM (Load volume: 0.5μL) |
| LOQ (10) | 0.628 μM (Load volume: 5μL) | 33.31 nM (Load volume: 0.5μL) |
